# Thermally Stable and Reusable Silica and Nano-Fructosome Encapsulated CalB Enzyme Particles for Rapid Enzymatic Hydrolysis and Acylation

**DOI:** 10.3390/ijms24129838

**Published:** 2023-06-07

**Authors:** Woo Young Jang, Jung Hoon Sohn, Jeong Ho Chang

**Affiliations:** 1 Korea Institute of Ceramic Engineering and Technology, Cheongju 28160, Republic of Korea; 2 Department of Materials Science & Engineering, Yonsei University, Seoul 03722, Republic of Korea; 3 Korea Research Institute of Bioscience and Biotechnology, Daejeon 34141, Republic of Korea

**Keywords:** thermal stability, reusability, silica, encapsulation, CalB enzyme, nano-fructosome

## Abstract

This study reports the preparation of silica-coated and nano-fructosome encapsulated *Candida antarctica* lipase B particles (CalB@NF@SiO_2_) and a demonstration of their enzymatic hydrolysis and acylation. CalB@NF@SiO_2_ particles were prepared as a function of TEOS concentration (3–100 mM). Their mean particle size was 185 nm by TEM. Enzymatic hydrolysis was performed to compare catalytic efficiencies of CalB@NF and CalB@NF@SiO_2_. The catalytic constants (K_m_, V_max_, and K_cat_) of CalB@NF and CalB@NF@SiO_2_ were calculated using the Michaelis–Menten equation and Lineweaver–Burk plot. Optimal stability of CalB@NF@SiO_2_ was found at pH 8 and a temperature of 35 °C. Moreover, CalB@NF@SiO_2_ particles were reused for seven cycles to evaluate their reusability. In addition, enzymatic synthesis of benzyl benzoate was demonstrated via an acylation reaction with benzoic anhydride. The efficiency of CalB@NF@SiO_2_ for converting benzoic anhydride to benzyl benzoate by the acylation reaction was 97%, indicating that benzoic anhydride was almost completely converted to benzyl benzoate. Consequently, CalB@NF@SiO_2_ particles are better than CalB@NF particles for enzymatic synthesis. In addition, they are reusable with high stability at optimal pH and temperature.

## 1. Introduction

Biocatalysts are used in various industries such as medicine, pharmaceuticals, cosmetics, and agriculture due to their substrate specificity, optical selectivity, and involvement in various chemical reactions within the body [1,2,3,4,5,6]. *Candida antarctica* lipase B (CalB) is a biocatalyst that belongs to the class of enzymes involved in catalyzing reactions. It has high activity and regioselectivity. Depending on reaction conditions, CalB can promote reactions such as hydrolysis and acidolysis [7,8,9,10,11]. CalB is expected to be a non-toxic and renewable energy source. It has the highest catalytic activity at neutral pH and 45 °C [12,13,14]. However, CalB is water soluble, which makes it highly sensitive to organic solvents, leading to reduced reaction efficiency [15,16]. Furthermore, it is difficult to store CalB for a long time. Its storage life is short, resulting in high costs in production and processing stages. In particular, it is difficult to maintain the structural stability of proteins during biochemical reactions, which can affect biological activity the most. Therefore, research on enzyme immobilization, which can complement shortcomings of CalB, is being actively conducted [17,18,19,20,21,22,23,24].

Enzyme immobilization, as a strategy to enhance the activity efficiency by increasing the stability of enzymes in solvents through various methods such as cross-linking, encapsulation, and surface modification, can contribute to improving the biochemical characteristics. Among the commercially available immobilized lipases, Novozym-435 is widely used in the fields of lipid modification and derivative production, polymer synthesis and degradation reactions, and biodiesel production due to its high stability [25,26]. However, despite being an excellent biocatalyst with broad applications, immobilized enzymes such as Novozym-435 suffer from issues such as modification of the solid support induced by the co-solvent, enzyme leaching, mechanical fragility, and high cost [25,27]. Therefore, efforts are being made to improve immobilized enzymes by modifying the problematic supports in order to address these challenges.

Levan, which is known as a fructose polymer, is a natural bio-polymer found in small amounts in some plants and microorganisms. Levan has a cationic property in an aqueous solution due to carboxyl and hydroxyl groups of its constituent substance fructose, contributing to its very stable shape. Levan is a functional material that is used in various industries such as functional cosmetics, agriculture, and industry because it is a water-soluble fructose polymer that melts not only at high temperatures, but also at low temperatures [28,29,30]. Therefore, levan-encapsulated enzymes or proteins have been studied and utilized to solve problems of reduced activities of proteinaceous substances such as enzymes. In addition, they are resistant to organic solvents [31,32,33,34]. Furthermore, we developed carboxymethyl levan (CML)-based nanocomposites dubbed ‘nanofructosome (NF)’ and demonstrated the effects of NF particles by entrapping lipase [35]. However, these levan-encapsulated enzyme complexes are still inherently denatured during long-term storage with poor acid and heat resistance, resulting in reduced catalytic activity. In addition, because it is a water-soluble fructose polymer that melts even at low temperatures, it is difficult to use it in continuous processes as an industrial catalyst. To overcome these limitations, more advanced immobilization techniques are needed.

Silica encapsulation technology is widely known as a method that can efficiently embed materials for industrial purposes [36,37,38,39,40]. The material encapsulated with silica is subordinated to the insoluble support, allowing multiple uses in a continuous process. As a result, it is heat resistant [36], and pH resistant [39], reducing sensitivity to environmental factors and contributing to high efficiency [40]. In addition, it enables long-term storage even at room temperature, effectively reducing the cost of industrial processes [38,40]. Therefore, encapsulation technology using silica has been demonstrated as an effective method for enzyme immobilization and continues to be actively researched [40,41,42,43,44]. Thus, it is expected that silica immobilization technology can also be applied to protein–levan complexes. Research is needed to effectively multi-use water-soluble enzymes in organic solvents.

In this study, the enzyme activity of native CalB immobilized via levan (Nano-fructose CalB, CalB@NF) to be used in organic solvents was stabilized under various environmental factors by encapsulating it using tetraethyl orthosilicate (TEOS), a silica precursor. CalB@NF@SiO_2_ particles were produced as a function of TEOS concentration ranging from 3–100 mM. The encapsulation morphology of CalB@NF@SiO_2_ was confirmed through TEM. Its catalytic activity and reusability were evaluated at various pH and temperature ranges compared to native CalB and CalB@NF. The enzyme amount of CalB@NF@SiO_2_ was confirmed through Bradford assay and TGA analysis. Catalytic parameters such as K_m_, V_max_, and K_cat_ were calculated using the Michaelis–Menten equation and a Lineweaver–Burk plot and confirmed through enzymatic hydrolysis using *p*-nitrophenyl butyrate (PNPB). Enzymatic synthesis was conducted through synthesis of benzyl benzoate using benzoic anhydride. The efficiency of converting benzoic anhydride to benzyl benzoate was calculated.

## 2. Results and Discussion

**Figure 1:**
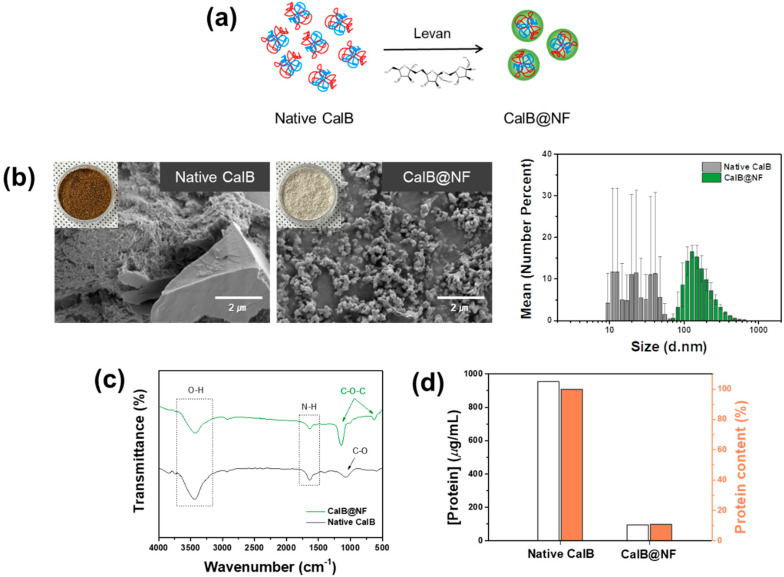
(**a**) CalB@NF preparation process with levan, (**b**) FE-SEM images (with particles size distribution), (**c**) FT-IR spectrum, and (**d**) protein quantification of native CalB and CalB@NF via Bradford assay.

Figure 1 shows a comparison of native CalB and CalB@NF. CalB@NF was inserted into levan, a fructose polymer, as shown in Figure 1a. CalB@NF was a complex formed by trapping protein inside a nanoparticle. The protein was simply trapped without forming a chemical bond with the levan nanoparticle. The morphological details and particles size of native CalB and CalB@NF are shown in Figure 1b. Both catalysts were in powder form, with native CalB having no specific shape, while CalB@NF particles were spherical with an average size of 150 nm. Moreover, the particle sizes of native CalB and CalB@NF followed a normal distribution, with d_50_ values of 42 nm and 156 nm, respectively. These results were obtained after dispersing the catalysts in distilled water using ultrasonic treatment for 15 min. The average particle size of CalB@NF, as confirmed by FE-SEM, was similar to the normal distribution. However, native CalB exhibited wide-range particle sizes in the normal distribution due to the ultrasonic treatment applied for dispersion. Figure 1c shows FT-IR spectra of native CalB and CalB@NF. Both samples showed common O–H stretching vibration at 3500 cm^−1^ and N–H bending at 1635 cm^−1^. In addition, anti-symmetric stretching of C–O–C and C–O–C aromatics bending was observed at 1150 cm^−1^ and 630 cm^−1^ for CalB@NF due to the chemical bond of levan. These results indicated that native CalB was successfully inserted into levan in CalB@NF via C–O–C bending of levan. Figure 1d shows amounts of CalB enzyme in native CalB and CalB@NF determined by the Bradford assay. Amounts of CalB enzyme for native CalB and CalB@NF at the same weight were 953.5 μg/mL and 94.7 μg/mL, respectively. Therefore, CalB@NF contained 10 times less protein than native CalB. To enable CalB@NF, in which the CalB enzyme was inserted into levan, to be used efficiently in industrial processes under various environmental conditions and for long-term storage at room temperature, silica encapsulation was performed to maintain stable catalytic activity.

**Figure 2:**
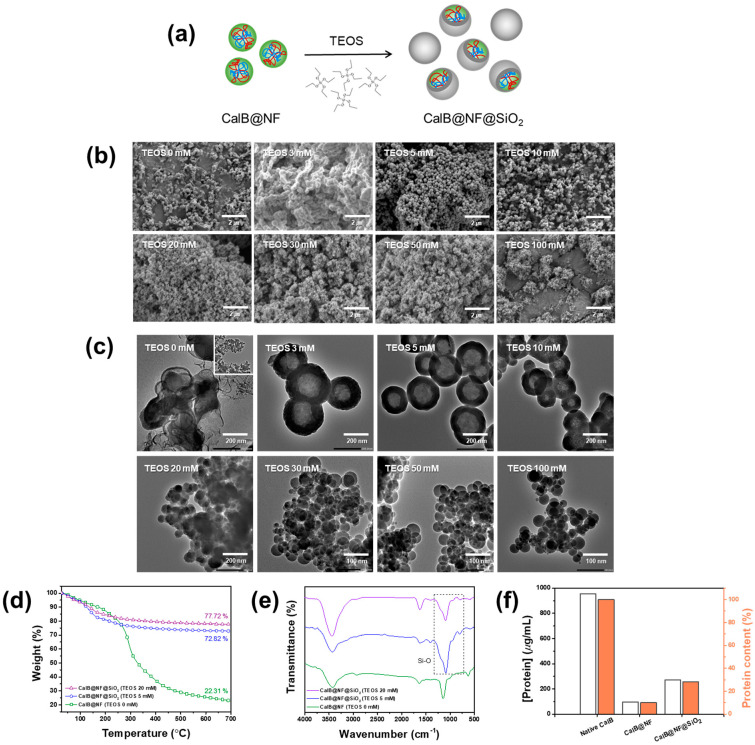
(**a**) Silica encapsulation process, (**b**,**c**) FE-SEM and TEM images of silica encapsulation on CalB@NF as a function of TEOS concentration, (**d**) TGA analysis, (**e**) FT-IR spectrum, (**f**) protein quantification of CalB@NF and CalB@NF@SiO_2_ (TEOS 5 mM) via Bradford assay.

CalB@NF@SiO_2_ was prepared by adjusting the TEOS concentration for CalB@NF in the range of 3–100 mM, as shown in Figure 2a. The morphology of the prepared CalB@NF@SiO_2_ was confirmed by FE-SEM and TEM for each TEOS concentration, as shown in Figure 2b,c. CalB@NF@SiO_2_ had a spherical shape. It became more uniform as the concentration of TEOS increased. Average sizes of particles prepared with TEOS concentrations of 0, 3, 5, 10, 20, 30, 50, and 100 mM were 150, 308, 186, 149, 35, 47, 52, and 66 nm, respectively. As shown in Figure 2c, a silica shell was obtained at TEOS concentrations of 3, 5, and 10 mM, but not obtained at a TEOS concentration of 0 mM, proving successful encapsulation. Moreover, only normal silica was observed at TEOS concentration of 20 mM or higher. As shown in Figure 2b, when TEOS was 20 mM or more, the average particle size was much smaller than that when the TEOS concentration was 0 mM, indicating that CalB@NF was not encapsulated. This result was because excessive TEOS concentration in the sol–gel reaction could cause silanols generated by hydrolysis to undergo a condensation reaction.

The resulting gel network was composed only of silica, forming spherical silica [45]. Therefore, the particle shape of CalB@NF@SiO_2_ had a confirmed silica shell and a uniform size, with 5 mM being the most optimal concentration of TEOS. Figure 2d shows amounts of CalB@NF in CalB@NF@SiO_2_ at various TEOS concentrations to investigate the amount of CalB@NF for each morphology change via TGA within a temperature range of 25–700 °C. The amounts of CalB@NF at TEOS 5 mM and 20 mM were 50.51% and 55.41%, respectively, compared to those at TEOS 0 mM. Figure 2e shows the FT-IR spectra of CalB@NF@SiO_2_. Characteristic vibrations of CalB@NF were observed during silica encapsulation. Asymmetric and symmetric stretching vibrations of Si–O–Si bonds were observed at 1080 cm^−1^, 970 cm^−1^, and 800 cm^−1^, indicating that CalB@NF was encapsulated by silica precursor TEOS. Figure 2f shows amounts of CalB enzyme in native CalB, CalB@NF, and CalB@NF@SiO_2_ (TEOS 5 mM) according to the Bradford assay. At the same weight, the amounts of CalB enzyme for native CalB, CalB@NF, and CalB@NF@SiO_2_ (TEOS 5 mM) were 953.5 μg/mL, 94.7 μg/mL, and 270.5 μg/mL, respectively. These results revealed that the amount of encapsulated CalB enzyme in CalB@NF@SiO_2_ was three times higher than that of CalB@NF. This result was supported by Figure 2d, where the amounts of CalB@NF and CalB@NF@SiO_2_ (TEOS 5 mM) were 22.31% and 72.82%, respectively, at 700 °C, with a ratio of approximately 1:3, which confirmed its reliability. Therefore, the ratio of native CalB:CalB@NF:CalB@NF@SiO_2_ (TEOS 5 mM) containing protein was 10:1:3.

**Figure 3:**
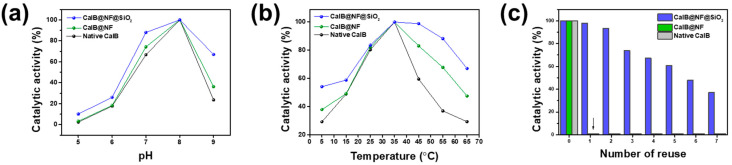
(**a**) Catalytic activity in different pH and (**b**) temperature (thermal stability), (**c**) reusability of native CalB, CalB@NF, and CalB@NF@SiO_2_ (TEOS 5 mM).

Figure 3 shows effects of encapsulation on catalytic activity, pH, thermal stability, and reusability. Catalytic activities of native CalB, CalB@NF, and CalB@NF@SiO_2_ (TEOS 5 mM) were evaluated through hydrolysis of PNPB. Figure 3a displays pH dependence of catalytic activities of native CalB, CalB@NF, and CalB@NF@SiO_2_ (TEOS 5 mM). All three samples exhibited optimum catalytic activities at pH 8, with reduced activities at other pH values. These results indicate that the catalytic activity is dependent on the enzyme’s active state at specific pH values. However, CalB@NF@SiO_2_ (TEOS 5 mM) exhibited 10–30% higher catalytic activity than native CalB and CalB@NF under all pH conditions, with pH stability provided by silica encapsulation. Figure 3b shows thermal stability of native CalB, CalB@NF, and CalB@NF@SiO_2_ (TEOS 5 mM). All three samples exhibited optimum catalytic activities at 35 °C, with reduced activities at other temperatures.

However, CalB@NF@SiO_2_ (TEOS 5 mM) exhibited up to 40% higher catalytic activity than native CalB and CalB@NF under all temperature conditions, with greater differences at higher temperatures. These results were due to silica interfering with protein heat transfer and the superior catalytic activity with thermal stability provided by insoluble silica encapsulation in an aqueous solution. Figure 3c presents reusability of native CalB, CalB@NF, and CalB@NF@SiO_2_ (TEOS 5 mM). Native CalB and CalB@NF could not be recovered for evaluation after one use. However, CalB@NF@SiO_2_ (TEOS 5 mM) could be recovered and used repeatedly, with reduced catalytic activity after each use. Therefore, enzyme immobilized in silica can be reused repeatedly, increasing the efficiency of the process and reducing costs through continuous use in industrial processes.

**Figure 4:**
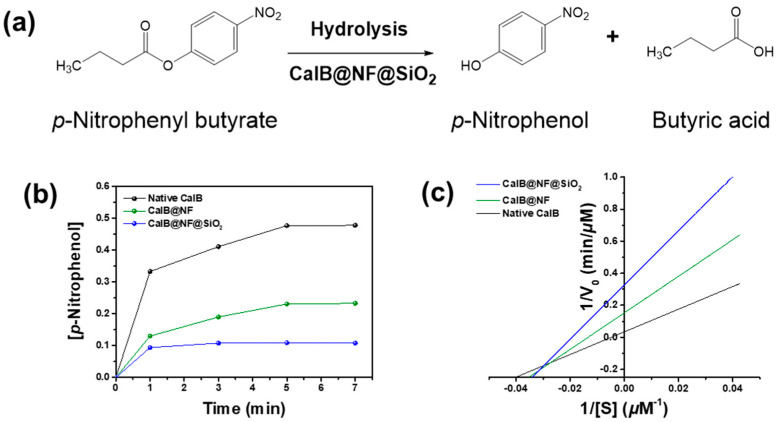
(**a**) Schemes of enzymatic hydrolysis with CalB@NF@SiO_2_ (TEOS 5 mM) against PNPB to PNP, (**b**) Absorption spectra of PNP as a function of reaction time, (**c**) Lineweaver–Burk plots of native CalB, CalB@NF, and CalB@NF@SiO_2_ (TEOS 5 mM).

Enzymatic hydrolysis was confirmed using native CalB, CalB@NF, and CalB@NF@SiO_2_ (TEOS 5 mM) with PNPB, as shown in Figure 4a. 

These results demonstrate that native CalB, CalB@NF, and CalB@NF@SiO_2_ (TEOS 5 mM) can participate in PNPB hydrolysis. Figure 4b shows concentrations of PNP generated during PNPB hydrolysis over time using native CalB, CalB@NF, and CalB@NF@SiO_2_ (TEOS 5 mM). Concentrations of PNP for all three samples did not increase after 5 min of reaction time, with an order of native CalB > CalB@NF > CalB@NF@SiO_2_ (TEOS 5 mM). A high concentration of PNP, the product of PNPB hydrolysis, implied that the reactant PNPB reacted more quickly or extensively with CalB in the catalyst. Thus, the higher the amount of CalB included in the catalyst, the faster the catalytic reaction occurs. The concentrations of CalB in native CalB, CalB@NF, and CalB@NF@SiO_2_ were 953.5 μg/mL, 94.7 μg/mL, and 270.5 μg/mL, respectively, as shown in Figure 2f. The efficiency of PNP production is as follows: native CalB > CalB@NF > CalB@NF@SiO_2_, which exhibits a partially different trend from the concentration of CalB in the catalyst. Despite the higher concentration of CalB in CalB@NF@SiO_2_ compared to CalB@NF, the PNP production efficiency is superior in CalB@NF. This indicates that the encapsulation of CalB by silica may obscure or block the active sites of CalB@NF@SiO_2_, resulting in lower hydrolysis efficiency compared to CalB@NF. However, in the case of native CalB, it has the highest concentration of CalB in the catalyst and no interfering substances on the active sites, leading to the highest hydrolysis efficiency. Figure 4c shows PNPB enzyme kinetics for native CalB, CalB@NF, and CalB@NF@SiO_2_ (TEOS 5 mM) obtained from the Lineweaver–Burk plot and Michaelis–Menten kinetics. Enzyme kinetic parameters were calculated. The results are shown in Table 1. The Michaelis–Menten constant (K_m_) represents an enzyme’s affinity for its substrate, with lower values indicating higher affinity and better binding between the enzyme and substrate. The turnover number (K_cat_) is a measure of an enzyme’s ability to convert substrate into product per unit time. The K_cat_/K_m_ ratio has been used as a measure of enzyme performance. K_m_ values were observed in the order of native CalB > CalB@NF > CalB@NF@SiO_2_, with CalB@NF@SiO_2_ showing the highest substrate affinity. V_max_ values were observed in the order of native CalB > CalB@NF > CalB@NF@SiO_2_, in contrast with substrate affinity results. In general, the reaction between a substrate and an enzyme proceeds faster when the enzyme has a higher affinity for the substrate. However, in this study, the high substrate affinity did not result in a correspondingly high reaction rate due to the number of hydroxyl groups on each catalyst surface and in the encapsulated layer. As the hydrolysis reaction occurred in an aqueous solution, the substrate was highly intimate with hydroxyl groups. Therefore, the surface of the silica-encapsulated CalB@NF@SiO_2_ synthesized using the sol–gel method contained a large number of hydroxyl groups. CalB@NF was produced using levan, which contained many hydroxyl groups, while native CalB only possessed hydroxyl groups that it originally had. Consequently, the number of hydroxyl groups on the surface of each catalyst was in the order of CalB@NF@SiO_2_ > CalB@NF > native CalB. Substrate affinity was also observed in the same order. Moreover, as the CalB@NF@SiO_2_ catalyst had an encapsulated layer that did not dissolve in the aqueous solution, the active site that had to directly bind to the substrate was obscured. Although CalB@NF was encapsulated with levan, which dissolved in the aqueous solution, it reacted with the substrate only to the extent that it possessed protein. Therefore, according to results shown in Figure 2f, the reaction rate was the highest for native CalB, which had the most protein, and the lowest for CalB@NF@SiO_2_, despite having a higher protein content than CalB@NF. This result was also confirmed in Figure 4b. The K_cat_ and K_cat_/K_m_ ratio related to the enzyme’s ability and efficiency were highest for CalB@NF, followed by CalB@NF@SiO_2_ and then native CalB. This result was calculated based on K_m_ and V_max_ values. CalB@NF, which had a high affinity for the substrate due to levan and reacted well with the substrate in the aqueous solution, was the most excellent. Therefore, CalB@NF was the best for enzymatic hydrolysis with the substrate. However, according to results shown in Figure 3, CalB@NF@SiO_2_ was the most convenient for use in harsh environments or continuous processes.

**Figure 5:**
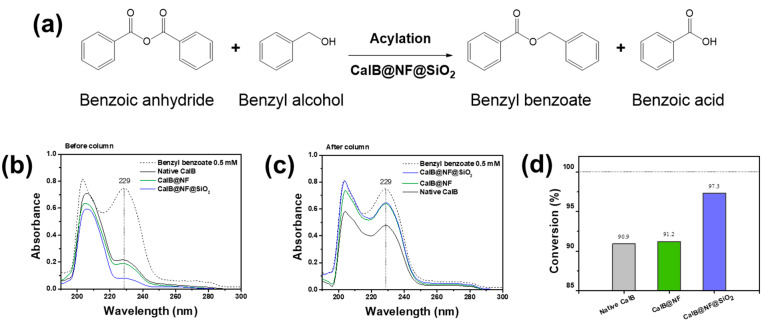
(**a**) Schemes of enzymatic synthesis for benzyl benzoate, (**b**,**c**) formed benzyl benzoate during enzymatic synthesis before and after column chromatography, (**d**) conversion efficiency of benzyl benzoate with native CalB, CalB@NF, and CalB@NF@SiO_2_ (TEOS 5 mM).

Figure 5 shows the process of synthesizing benzyl benzoate through enzymatic synthesis and its conversion efficiency. As shown in Figure 5a, enzymatic synthesis of benzyl benzoate using CalB@NF@SiO_2_ (TEOS 5 mM) involves reactions using benzoic anhydride as a reactant. In the reaction, benzoic anhydride has two acyl donors, leading to the generation of not only benzyl benzoate (first acyl donor), but also benzoic acid (second acyl donor) when reacting with benzyl alcohol [39,46]. Therefore, benzoic anhydride is suitable for synthesizing benzyl benzoate. According to this phenomenon, benzyl benzoate conversion efficiency was evaluated using native CalB, CalB@NF, and CalB@NF@SiO_2_ (TEOS 5 mM). Figure 5b shows results of UV-vis analysis of synthesized benzyl benzoate. Benzyl benzoate showed absorbance at 229 nm. However, it was difficult to distinguish because it was hindered by benzyl alcohol or benzoic acid that remained in the synthesis process. Therefore, it was separated and purified through column chromatography and analyzed for UV-vis (Figure 5c). As a result, native CalB, CalB@NF, and CalB@NF@SiO_2_ (TEOS 5 mM) all showed benzyl benzoate at 229 nm. Therefore, native CalB, CalB@NF, and CalB@NF@SiO_2_ (TEOS 5 mM) were demonstrated during enzymatic synthesis of benzyl benzoate in this process. Figure 5d shows the conversion efficiency of benzoic anhydride to benzyl benzoate using native CalB, CalB@NF, and CalB@NF@SiO_2_ (TEOS 5 mM). The conversion efficiency was calculated according to the following equation:

$$Conversion\left(\%\right)=\frac{C_{i}-C_{f}}{C_{i}}\times 100$$

In this equation, *C_i_* and *C_f_* were the initial and final concentrations of benzoic anhydride before and after enzymatic synthesis, respectively. The conversion efficiency from benzoic anhydride to benzyl benzoate was calculated to be 90.9%, 91.2%, and 97.3% for native CalB, CalB@NF, and CalB@NF@SiO_2_ (TEOS 5 mM), respectively. Although conversion efficiencies of native CalB and CalB@NF were similar, that of CalB@NF@SiO_2_ (TEOS 5 mM) was found to be almost 100%. This result suggests that silica-encapsulated CalB@NF@SiO_2_ (TEOS 5 mM) can prevent a decrease in catalytic activity in organic solvents. Therefore, protein immobilization through silica provides higher stability in organic solvents, resulting in increased catalytic efficiency.

## 3. Materials and Methods

### 3.1. Materials

*Candida antarctica* lipase B (Native CalB) and CalB@NF were obtained from Korea Research Institute of Bioscience and Biotechnology (33 kDa, Daejeon, Republic of Korea) used in encapsulation. Oleic acid, Iepal CO-520, *p*-nitrophenyl butyrate (98%, PNPB), bovine serum albumin, tris base, phosphate-buffered saline (PBS, St. Louis, MO, USA), Coomassie brilliant blue G-250 (Gilingham, Dorset, UK), phosphoric acid (Buchs, Switzerland), Tetraethyl orthosilicate (99%, TEOS, Beijing, China), benzoic anhydride (St. Quentin Fallavier, France), and benzyl alcohol (Taufkirchen, Germany) were purchased from Sigma-Aldrich. Cyclohexane, ammonia solution (28–30%), ethanol (99%), methanol, sodium acetate, glacial acetic acid, hydrochloric acid, and *n*-hexane were purchased from Daejung (Siheung, Republic of Korea). Ethyl ether was provided by J.T.Baker (Philipsburg, NJ, USA).

### 3.2. Preparation of Silica Encapsulated CalB@NF (CalB@NF@SiO_2_)

CalB@NF@SiO_2_ particles were prepared using a sol–gel method [44]. Oleic acid (2 mL, solution A), Igepal CO-520 (24 g, solution B), and CalB@NF (90 mg, solution C) were mixed with cyclohexane (100, 300, and 20 mL, respectively). After adding solution A and solution C to solution B, TEOS of various concentrations (3–100 mM) was added drop-wise and stirred for 1 h. Then, 163 mL of ammonia solution was added and stirred for 20 h. After stirring was complete, 325 mL of methanol was added. After precipitate formation was confirmed, the supernatant was removed. The precipitate was washed three times with *n*-hexane and completely vacuum dried at room temperature. The amount of entrapped enzyme was determined via Bradford assay using a protein reagent [47,48].

### 3.3. pH and Thermal Stability of Native CalB, CalB@NF, and CalB@NF@SiO_2_

Effects of pH and temperature on stability of native CalB, CalB@NF, and CalB@NF@SiO_2_ were determined based on their catalytic activities using PNPB at various pH (0.1 M of sodium acetate for pH 5–6 and 0.1 M of tris-HCl for pH 7–9) and temperature (5–65 °C) conditions. After 0.1 mg/mL of native CalB, CalB@NF, or CalB@NF@SiO_2_ was added to the substrate solution, catalytic activities were calculated at optimum pH and temperature conditions.

### 3.4. Reusability of Native CalB, CalB@NF, and CalB@NF@SiO_2_

Reusability of native CalB, CalB@NF, and CalB@NF@SiO_2_ was evaluated in repeated cycles with PNPB. First, 0.1 mg/mL of native CalB, CalB@NF, or CalB@NF@SiO_2_ was added into the substrate solution. Nnative CalB, CalB@NF, and CalB@NF@SiO_2_ were then recovered from reaction medium by centrifugation at 15,000 rpm for 1 min and washed with 0.1 M of tris-HCl buffer (pH 7.4) to remove any residual substrate. The process was evaluated up to 7 cycles to determine the reusability of each catalyst. The absorbance was measured at 400 nm by UV-Visible spectrophotometry (SCINCO, Seoul, Republic of Korea).

### 3.5. Enzymatic Hydrolysis for p-Nitrophenyl Butyrate with Native CalB, CalB@NF, and CalB@NF@SiO_2_

Enzymatic hydrolysis against PNPB was achieved with native CalB, CalB@NF, or CalB@NF@SiO_2_. PNPB solution at 0.1 M was prepared and diluted to different concentrations (0.00625–1 mM) with 0.1 M of tris-HCl buffer (pH 7.4). After 0.1 mg/mL of native CalB, CalB@NF, or CalB@NF@SiO_2_ was added into substrate solution at room temperature, the mixture was centrifuged at 15,000 rpm for 1 min. The absorbance of the product, *p*-nitrophenol (PNP), was measured at 400 nm using UV-Visible spectrophotometry. Catalytic parameters such as the Michaelis–Menten constant (K_m_), maximum reaction velocity (V_max_), and turnover value (K_cat_) were calculated for native CalB, CalB@NF, and CalB@NF@SiO_2_.

### 3.6. Enzymatic Synthesis for Benzyl Benzoate with Native CalB, CalB@NF, and CalB@NF@SiO_2_

Enzymatic synthesis of benzyl benzoate proceeded through an acylation reaction by mixing 10 mL of benzoic anhydride and 2.26 g of benzyl alcohol. After 0.5 g of native CalB, CalB@NF, or CalB@NF@SiO_2_ was added, the mixture was incubated at 50 °C for 24 h. After the reaction was completed, the supernatant was separated via centrifugation at 2500 rpm for 5 min. Separated supernatants were then subjected to column chromatography. The solvent was evaporated using a rotary evaporator after thin-layer chromatography (TLC) analysis. The production of benzyl benzoate was confirmed by measuring absorbance at 229 nm using UV-Visible spectrophotometry.

### 3.7. Characterizations

Transmitted morphological details of the prepared CalB@NF@SiO_2_ according to TEOS concentration were evaluated with a JEM-2100Plus transmission electron microscope (TEM) (JEOL, Tokyo, Japan) at an accelerating voltage of 200 kV. Overall morphological details were analyzed using TESCAN MIRA3 field emission scanning electron microscopy (FE-SEM) (TESCAN, Brno, Czech Republic) operated at 2 kV. The particle sizes of the native CalB and CalB@NF powder were measured via dynamic light scattering (DLS) using a Nano-ZS (Malvern Panalytical, Malvern, UK). To evaluate the amount of enzyme encapsulated in CalB@NF@SiO_2_, thermogravimetric analysis (TGA) at 700 °C (10 °C/min) in a nitrogen atmosphere was performed with a Q600 TA instrument (Waters, Milford, MA, USA). Fourier transform infrared (FT-IR) was performed with the KBr method using Frontier (PerkinElmer, Waltham, MA, USA) for characterization of CalB@NF@SiO_2_. UV-Visible spectrophotometry was performed using a Mega 900 (SCINCO) instrument at 190–500 nm. The solution obtained from the enzymatic synthesis of benzyl benzoate with CalB@NF@SiO_2_ was purified through column chromatography. Column chromatography was performed on silica gel with a pore size of 60 Å and a particle size of 32–63 nm. After purification, the reaction was confirmed via thin chromatography (TLC) visualized with 240 nm UV light on Merck silica gel 60 F254 plates (Merck & Co., Rahway, NJ, USA).

## 4. Conclusions

CalB@NF@SiO_2_ was prepared as a function of TEOS concentration ranging from 3 to 100 mM. The resulting CalB@NF@SiO_2_ showed a uniformly spherical shape depending on the TEOS concentration, although particle size and shape varied. The morphology analyzed through TEM revealed the presence of a silica shell at TEOS concentrations of 3–10 mM, while only pure silica was observed at concentrations above 20 mM. CalB@NF@SiO_2_ prepared as a function of various TEOS concentrations exhibited the most uniform shape at a TEOS concentration of 5 mM, which demonstrated stability under various pH and temperature conditions. Furthermore, it was confirmed that CalB@NF@SiO_2_, but not native CalB or CalB@NF, could be used repeatedly. In addition, enzymatic hydrolysis of PNPB and enzymatic synthesis of benzyl benzoate were proven. Enzymatic hydrolysis showed that PNPB was converted to PNP via Lineweaver–Burk plot and Michaelis–Menten kinetics. Enzymatic synthesis showed that the conversion efficiency of benzyl benzoate corresponding to CalB@NF@SiO_2_ (TEOS 5 mM) was 97.3%, indicating that almost all benzoic anhydride was converted to benzyl benzoate. Therefore, CalB@NF@SiO_2_ is more stable in aqueous solutions with respect to pH and temperature than native CalB and CalB@NF. In addition, it can be reused. It is expected that this will result in significant cost savings, as it exhibits minimal changes in response to environmental variations and can be employed in continuous processes.

## Figures and Tables

**Table 1:** Michaelis–Menten parameters of native CalB, CalB@NF, and CalB@NF@SiO_2_ (TEOS 5 mM) for hydrolysis of PNPB.

	K_m_ (mM)	V_max_ (μM∙min^−1^)	K_cat_ (min^−1^)	K_cat_/K_m_ (μM^−1^∙min^−1^)
Native CalB	0.204	28.74	9484	46,709
CalB@NF	0.074	11.41	21,473	289,300
CalB@NF@SiO_2_	0.051	3.05	10,065	195,575
